# Targeted sequencing of tonsillar and base of tongue cancer and human papillomavirus positive unknown primary of the head and neck reveals prognostic effects of mutated FGFR3

**DOI:** 10.18632/oncotarget.15240

**Published:** 2017-02-09

**Authors:** Cinzia Bersani, Lars Sivars, Linnea Haeggblom, Sebastian DiLorenzo, Michael Mints, Ährlund-Richter Andreas, Nikolaos Tertipis, Eva Munck-Wikland, Anders Näsman, Torbjörn Ramqvist, Tina Dalianis

**Affiliations:** ^1^ Department of Oncology-Pathology, Karolinska Institutet, Stockholm, Sweden; ^2^ Department of Medical Sciences, Uppsala University, Uppsala, Sweden; ^3^ National Bioinformatics Infrastructure Sweden, Science for Life Laboratory, Uppsala University, Uppsala, Sweden; ^4^ Department of Medicine, Karolinska Institutet, Stockholm, Sweden; ^5^ Department of Surgical and Perioperative Sciences, Umeå University, Umeå, Sweden; ^6^ Department of Clinical Science and Technology, Karolinska Institutet, Stockholm, Sweden; ^7^ Department of Oto-Rhino-Laryngology, Head and Neck Surgery, Karolinska University Hospital, Stockholm, Sweden

**Keywords:** HPV, tonsillar cancer, base of tongue cancer, cancer of unknown primary of the head and neck region, FGFR3 mutation

## Abstract

**BACKGROUND:**

Human papillomavirus positive (HPV^+^) tonsillar cancer (TSCC), base of tongue cancer (BOTSCC) and unknown primary cancer of the head and neck (HNCUP) have better outcome than corresponding HPV^−^ cancers. To find predictive markers for response to treatment, and correlations and differences in mutated oncogenes and suppressor genes between HPV^+^ TSCC/BOTSSCC and HPV^+^ HNCUP and HPV^−^ TSCC/BOTSCC targeted next-generation sequencing was performed of frequently mutated regions in 50 cancer related genes.

**PATIENTS AND METHODS:**

DNA from 348 TSCC/BOTSCC and 20 HNCUP from patients diagnosed 2000-2011, was sequenced by the Ion Proton sequencing platform using the Ion AmpliSeq Cancer Hotspot Panel v2 to identify frequently mutated regions in 50 cancer related genes. Ion Torrent Variant Caller software was used to call variants.

**RESULTS:**

279 HPV^+^ TSCC/BOTSCC, 46 HPV^−^ TSCC/BOTSCC and 19 HPV^+^ HNCUP samples qualified for further analysis. Mutations/tumor were fewer in HPV^+^ TSCC/BOTSCC and HNCUP, compared to HPV^−^ tumors (0.92 *vs*. 1.32 *vs*. 1.68). Differences in mutation frequency of TP53 and PIK3CA were found between HPV^+^ TSCC/BOTSCC and HNCUP and HPV^−^ TSCC/BOTSCC. In HPV^+^ TSCC/BOTSCC presence of FGFR3 mutations correlated to worse prognosis. Other correlations to survival within the groups were not disclosed.

**CONCLUSIONS:**

In HPV^+^ TSCC/BOTSCC mutation of PIK3CA was most frequently observed, while TP53 mutations dominated in HPV^−^ TSCC/BOTSCC. In HPV^+^ TSCC/BOTSCC and HNCUP, mutations/tumor were similar in frequency and fewer compared to that in HPV^−^ TSCC/BOTSCC. Notably, FGFR3 mutations in HPV^+^ TSCC/BOTSCC indicated worse prognosis.

## INTRODUCTION

Human papillomavirus positive (HPV^+^) tonsillar squamous cell carcinoma (TSCC) and base of tongue squamous cell carcinoma (BOTSCC) and head neck unknown primary cancer (HNCUP) have a much better clinical outcome than the corresponding HPV^−^ tumors and some other types of head and neck squamous cell carcinoma (HNSCC) [1–6]. Furthermore, the incidences of TSCC and BOTSCC, the two subtypes of oropharyngeal squamous cell carcinoma (OPSCC), where HPV is most frequently found, have increased considerably in many Western countries [5–14]. HNSCC therapy is today often aggressive with radiotherapy, induction/concomitant chemoradiotherapy, targeted therapy and surgery. However, most HPV^+^ TSCC/BOTSCC and HNCUP patients do not need intensified treatment, and the possibility to de-escalate, or better tailor treatment, while maintaining survival and reducing treatment-related morbidity would be of significant benefit [5].

To better individualize treatments, several efforts have been made to identify additional predictive markers [15–23]. Age, stage, smoking, presence of HPV16 E2 mRNA, absent/low HLA class I expression, CD44, LMP10 expression, high LRIG1 expression, absence of HLA-A*02, high CD8^+^ tumor infiltrating lymphocyte (TIL) counts and CD98 have been proposed as such markers in HPV^+^ TSCC and BOTSCC [15–25]. One by one, or in combination in mathematical models, they can identify 20-40% of patients with up to > 95% probability to have a 3-year disease free survival in HPV^+^ TSCC/BOTSCC [24]. However, additional markers are necessary to distinguish a larger proportion of patients with tumors with a very high probability to respond easily and thoroughly to therapy. Furthermore, the search for new markers may very well lead to the disclosure of specific proteins and/or mutations in genes that could be targeted by existing or novel targeted therapies.

In the case of HPV^+^ HNCUP, since its survival after treatment is similar to that of HPV^+^ TSCC/BOTSCC, one hypothesis put forward is that it may originate from an HPV^+^ TSCC or BOTSCC [4, 26–28]. Nevertheless, so far there is limited evidence of the exact nature of HPV^+^ HNCUP and there are studies that have not found the same impact of HPV on survival [29].

To find more predictive markers for response to therapy for HPV^+^ TSCC/BOTSCC, and similarities and differences of mutated genes compared to HPV^+^ HNCUP and HPV^−^ TSCC/BOTSCC, as well as potential targets for new therapies, next-generation sequencing (NGS) of hotspot mutations in 50 cancer related genes, was done. Examining TP53 and PIK3CA was of special interest, since these genes have previously been reported to be differentially mutated in HPV^+^ and HPV^−^ OPSCC and their impact on outcome at the genomic level has not been studied extensively in HPV^+^ and HPV^−^ OPSCC [30].

## RESULTS

### Next-generation sequencing, calling and filtering of variants

DNA from 368 formalin-fixed paraffin-embedded (FFPE) tumor samples including 297 HPV^+^ and 51 HPV^−^ TSCC/BOTSCC and 20 HPV^+^ HNCUP was analyzed using the Ion AmpliSeq Cancer Hotspot Panel v2 (CHPv2) covering hotspot regions from 50 oncogenes and tumor suppressor genes. After excluding 24 samples due to poor DNA quality, 279 HPV^+^, 46 HPV^−^ TSCC/BOTSCC and 19 HPV^+^ HNCUP samples remained for further analysis. Patient and tumor characteristics for the included samples are presented in Table 1 (TSCC and BOTSCC) and Table 2 (HNCUP) and in more detail in the material and methods section. Of the 279 HPV DNA positive TSCC/BOTSCC samples, 255 were HPV16 positive, while the remaining were HPV33, 35, 56, 58 or 59 positive and all but one overexpressed p16. All HNCUP samples were HPV16 positive and all but two overexpressed p16.

**Table 1:** Patient and TSCC/BOTSCC characteristics

Patient and tumor characteristics		HPV+TSCC/BOTSCC (*N*= 279)		HPV-TSCC/BOTSCC (*N*= 46)		All TSCC/BOTSCC (*N*= 325)	
		*N*	%	*N*	%	*N*	%
Age	*Mean (years)*	60.0		63.0		60	
	*Median (years)*	59		62		60.0	
	*Range (years)*	30-84		46-85		30-84	
Diagnose	*malignant neoplasm of the base of tongue (C01.9)*	73	26%	14	30%	87	27%
	*malignant neoplasm of the tonsil (C09.0-9)*	206	74%	37	80%	243	75%
Sex	*female*	65	23%	9	20%	74	23%
	*male*	214	77%	37	80%	251	77%
Tumour differentiation	*poorly*	180	65%	29	63%	209	64%
	*moderatley*	75	27%	12	26%	87	27%
	*well*	16	6%	5	11%	21	6%
	*undefined*	8	3%	0	0%	8	2%
Tumour size	*T1*	78	28%	6	13%	84	26%
	*T2*	107	38%	12	26%	119	37%
	*T3*	52	19%	14	30%	66	20%
	*T4*	42	15%	14	30%	56	17%
Nodal disease	*N0*	37	13%	17	37%	54	17%
	*N1*	61	22%	3	7%	64	20%
	*N2a*	37	13%	4	9%	41	13%
	*N2b*	109	39%	12	26%	121	37%
	*N2c*	25	9%	7	15%	32	10%
	*N3*	8	3%	3	7%	11	3%
	*NX*	2	1%	0	0%	2	1%
Distant metastasis	*M0*	275	99%	46	100%	321	99%
	*M1*	2	1%	0	0%	2	1%
	*MX*	2	1%	0	0%	2	1%
Tumour Stage	*I*	4	1%	5	11%	9	3%
	*II*	18	6%	3	7%	21	6%
	*III*	65	23%	9	20%	74	23%
	*IVa*	178	64%	25	54%	203	62%
	*IVb*	9	3%	4	9%	13	4%
	*IVc*	2	1%	0	0%	2	1%
	*Unknown*	3	1%	0	0%	3	1%
Treatment	*Induction chemotherapy and radiation*	22	8%	7	15%	29	9%
		93	33%	5	11%	98	30%
	*Radiation*	115	41%	24	52%	139	43%
		47	17%	7	15%	54	17%
	*Palliative*	2	1%	3	7%	5	2%
Brachytherapy boost	*Not administered*	207	74%	33	72%	240	74%
	*Administered*	70	25%	10	22%	80	25%
Concomittant Cetuximab	*Not administered*	225	81%	40	87%	265	82%
	*Administered*	52	19%	3	7%	55	17%
Smoking	*Never*	105	38%	4	9%	109	34%
	*Former (>15 years ago)*	54	19%	1	2%	55	17%
	*Former (<15 years ago)*	53	19%	4	9%	57	18%
	*Current upon diagnosis*	67	24%	35	76%	103	32%
	*Unknown*	0	0%	2	4%	2	1%

**Table 2:** Patient and HPV+ HNCUP characteristics

Patient and tumor characteristics		HNCUP patients (*N*= 19)	
		*N*	%
Age	*Mean (years)*	63.1	
	*Median (years)*	65	
	*Range (years)*	36-91	
Sex	*female*	4	21%
	*male*	15	79%
Nodal disease	*N0*	0	0%
	*N1*	7	37%
	*N2a*	2	11%
	*N2b*	6	32%
	*N2c*	2	11%
	*N3*	2	11%
	*NX*	0	0%
Distant metastasis	*M0*	19	100%
	*M1*	0	0%
	*MX*	0	0%
Smoking	*Never*	4	21%
	*Former (>15 years ago)*		
	*Former (<15 years ago)*		
	*Current upon diagnosis*		

Variants were called using the Torrent Variant Caller (TVC) version 5.0 and after filtering for > 5% allele frequency and an allele coverage of > 100 the remaining variants were filtered against several population genome databases as specified in the materials and methods section.

### Variants in HPV^+^ and HPV^−^ TSCC/BOTSCC exhibit differences based on HPV status

After filtering, 337 variants remained in 325 TSCC/BOTSCC distributed in the different target genes as presented in Table 3. In HPV^+^ TSCC/BOTSCC, the most common variants were PIK3CA, TP53, FGFR3, FBXW7, PTEN and CDKN2A, while in HPV^−^ TSCC/BOTSCC mutations were most frequently found in TP53, PIK3CA, IDH2, ABL1, BRAF, CDKN2A, EGFR, NOTCH1 and PTPN11. Significant differences in frequency of variants between the two groups are shown in Table 3. Variants were more common in HPV^−^ than in HPV^+^ TSCC/BOTSCC with 0.92 *vs*. 1.68 variant/tumor respectively and were detected in 48.7% (136/279) of HPV^+^ and 74.5% (35/46) of the HPV^−^ cases (*p* = 0.0007). This difference was mainly due to the difference in TP53 variants (9.3% *vs*. 63.8% for HPV^+^ *vs*. HPV^−^ TSCC/BOTSCC). Non-TP53 mutations tended to be more frequent in HPV^+^ compared to HPV^−^ TSCC/BOTSCC (46.6% *vs*. 30.4%, *p* = 0.0538). PIK3CA was the most frequently mutated gene in HPV^+^ TSCC/BOTSCC and was significantly more frequently mutated in HPV^+^ than in HPV^−^ TSCC/BOTSCC (20.1% *vs*. 6.4%). As presented in Table 3, two more genes IDH2 and NOTCH1, also showed significant differences in numbers of variants between HPV^+^ and HPV^−^ TSCC/BOTSCC, but in these cases the numbers of affected tumors were small and the data should be treated with caution. Differences between the two groups are visualized in Supplementary Figure S1.

**Table 3:** Frequency of variants in TSCC/BOTSCC and HNCUP

Tumor	HPV+ TSCC/BOTSCC (n=279)				HPV- TSCC/BOTSCC (N=46)					HPV+ HNCUP (n=19)			
Gene	Total no of variants	Variants/ tumor	Tumors with variant gene	% tumors with variant gene	Total no of variants	Variants/tumor	Tumors with variant gene	% tumors with variant gene	^1^*p*-values	Total no of variants	Variants/ tumor	Tumors with variant gene	% tumors with variant gene
ABL1	1	0.004	1	0.4%	2	0.04	2	4.3%		0	0.000	0	0.0%
AKT1	3	0.011	3	1.1%	0	0.00	0	0.0%		0	0.000	0	0.0%
ALK	1	0.004	1	0.4%	0	0.00	0	0.0%		0	0.000	0	0.0%
APC	2	0.007	2	0.7%	0	0.00	0	0.0%		1	0.053	1	5.3%
ATM	7	0.025	7	2.5%	1	0.02	1	2.1%		0	0.000	0	0.0%
BRAF	3	0.011	3	1.1%	2	0.04	2	4.3%		0	0.000	0	0.0%
CDH1	1	0.004	1	0.4%	1	0.02	1	2.1%		0	0.000	0	0.0%
CDKN2A	13	0.047	12	4.3%	3	0.06	2	4.3%		4	0.211	3	15.8%
CSF1R	0	0.000	0	0.0%	0	0.00	0	0.0%		0	0.000	0	0.0%
CTNNB1	4	0.014	3	1.1%	0	0.00	0	0.0%		0	0.000	0	0.0%
EGFR	11	0.039	7	2.5%	3	0.06	2	4.3%		2	0.105	2	10.5%
ERBB2	0	0.000	0	0.0%	0	0.00	0	0.0%		0	0.000	0	0.0%
ERBB4	0	0.000	0	0.0%	1	0.02	1	2.1%		0	0.000	0	0.0%
EZH2	0	0.000	0	0.0%	0	0.00	0	0.0%		0	0.000	0	0.0%
FBXW7	18	0.065	18	6.5%	0	0.00	0	0.0%		1	0.053	1	5.3%
FGFR1	1	0.004	1	0.4%	1	0.02	1	2.1%		0	0.000	0	0.0%
FGFR2	0	0.000	0	0.0%	0	0.00	0	0.0%		0	0.000	0	0.0%
FGFR3	22	0.079	20	7.2%	1	0.02	1	2.1%		0	0.000	0	0.0%
FLT3	0	0.000	0	0.0%	1	0.02	1	2.1%		0	0.000	0	0.0%
GNA11	0	0.000	0	0.0%	0	0.00	0	0.0%		0	0.000	0	0.0%
GNAQ	0	0.000	0	0.0%	0	0.00	0	0.0%		0	0.000	0	0.0%
GNAS	0	0.000	0	0.0%	0	0.00	0	0.0%		0	0.000	0	0.0%
HNF1A	0	0.000	0	0.0%	1	0.02	1	2.1%		0	0.000	0	0.0%
HRAS	6	0.022	5	1.8%	0	0.00	0	0.0%		0	0.000	0	0.0%
IDH1	0	0.000	0	0.0%	1	0.02	1	2.1%		0	0.000	0	0.0%
IDH2	1	0.004	1	0.4%	3	0.06	3	6.4%	0.0098	2	0.105	2	10.5%
JAK2	0	0.000	0	0.0%	1	0.02	1	2.1%		0	0.000	0	0.0%
JAK3	6	0.022	6	2.2%	0	0.00	0	0.0%		0	0.000	0	0.0%
KDR	1	0.004	1	0.4%	1	0.02	1	2.1%		0	0.000	0	0.0%
KIT	1	0.004	1	0.4%	1	0.02	1	2.1%		0	0.000	0	0.0%
KRAS	9	0.032	9	3.2%	0	0.00	0	0.0%		0	0.000	0	0.0%
MET	4	0.014	4	1.4%	1	0.02	1	2.1%		0	0.000	0	0.0%
MLH1	0	0.000	0	0.0%	0	0.00	0	0.0%		0	0.000	0	0.0%
MPL	0	0.000	0	0.0%	1	0.02	1	2.1%		0	0.000	0	0.0%
NOTCH1	0	0.000	0	0.0%	2	0.04	2	4.3%	0.0197	0	0.000	0	0.0%
NPM1	0	0.000	0	0.0%	0	0.00	0	0.0%		0	0.000	0	0.0%
NRAS	4	0.014	4	1.4%	0	0.00	0	0.0%		0	0.000	0	0.0%
PDGFRA	0	0.000	0	0.0%	0	0.00	0	0.0%		0	0.000	0	0.0%
PIK3CA	58	0.208	56	20.1%	3	0.06	3	6.4%	0.0240	3	0.158	3	15.8%
PTEN	16	0.057	15	5.4%	1	0.02	1	2.1%		0	0.000	0	0.0%
PTPN11	1	0.004	1	0.4%	3	0.06	2	4.3%		0	0.000	0	0.0%
RB1	3	0.011	3	1.1%	0	0.00	0	0.0%		0	0.000	0	0.0%
RET	0	0.000	0	0.0%	0	0.00	0	0.0%		0	0.000	0	0.0%
SMAD4	4	0.014	5	1.8%	2	0.04	1	2.1%		0	0.000	0	0.0%
SMARCB1	4	0.014	7	2.5%	0	0.00	0	0.0%		0	0.000	0	0.0%
SMO	2	0.007	2	0.7%	0	0.00	0	0.0%		0	0.000	0	0.0%
SRC	0	0.000	0	0.0%	1	0.02	1	2.1%		0	0.000	0	0.0%
STK11	5	0.018	5	1.8%	0	0.00	0	0.0%		0	0.000	0	0.0%
TP53	44	0.158	26	9.3%	40	0.85	30	63.8%	<0.0001	11	0.579	5	26.3%
VHL	2	0.007	2	0.7%	1	0.02	1	2.1%		1	0.053	1	5.3%
All genes	258	0.925	136	48.7%	79	1.68	35	74.5%	0.0007	25	1.316	9	47.4%

^1^*p*-values for number of tumors with variants in HPV positive *vs*. negative TSCC/BOTSCC as evaluated by chi^2^ or Fisher´s exact test.

Only *p*-values <0.05 are shown

**Figure 1:**
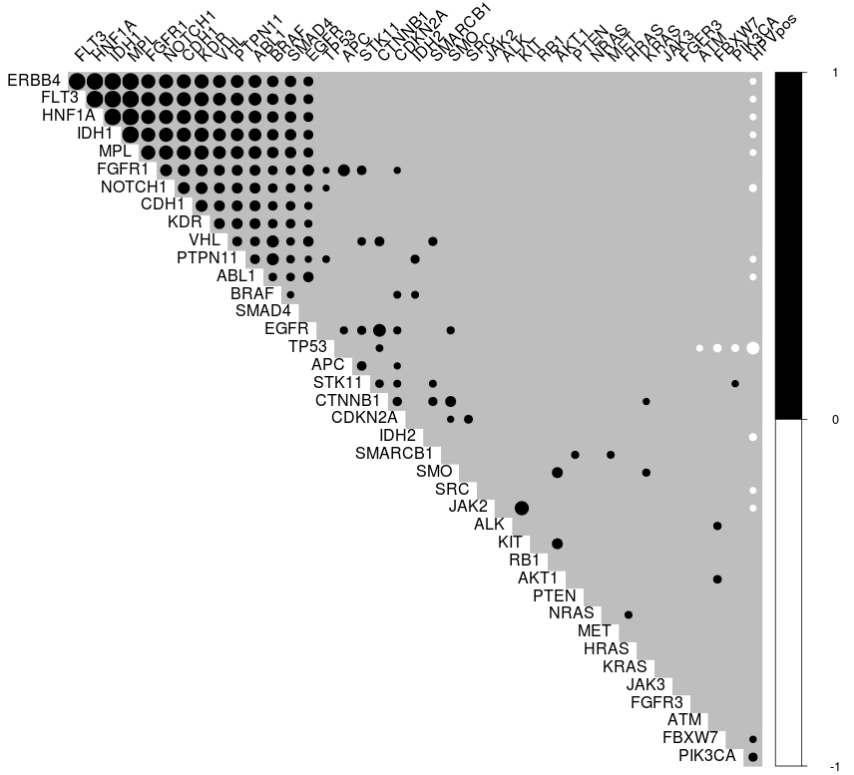
Plot of correlations between gene mutations and HPV status Only significant correlations (*p* < 0.05) were included. Black circles indicate positive correlations, while white indicate negative correlations. The size of the circle indicates the Spearman correlation coefficient. The black block in the upper left corner shows a number of genes with strong inter-correlations, which mostly have an inverse relationship with HPV status. HPV is negatively associated with many mutations, most strongly TP53, and only positively correlated with PIK3CA and FBXW7 mutations.

Spearman correlations were calculated between the presence of any mutation in the genes analyzed and HPV status. It was found that a number of genes (ERBB4, FLT3, HNF1A, IDH1 etc.) are mutated in highly correlated blocks, and that most of these mutations are absent in patients with HPV^+^ TSCC/BOTSCC. TP53 mutations had the strongest inverse correlation with HPV^+^ TSCC/BOTSCC, while PIK3CA and FBXW7 showed positive correlations (Figure 1).

### Variants in HPV^+^ HNCUP

In the 19 HPV^+^ HNCUP, 25 variants (1.32 variants/tumor) were observed in TP53, PIK3CA, CDKN2A, EGFR, IDH2, FBXW7 and VHL, for details see Table 3. In total, 47% (9/19) of the tumors contained variants, which was similar to the frequency in HPV^+^ TSCC/BOTSCC (48.7%), but less than in HPV^−^ TSCC/BOTSCC (74.5%) (*p* = 0.04). TP53 was more often mutated in HPV^+^ HNCUP than in HPV^+^ TSCC/BOTSCC (*p* = 0.0345), but less often than in HPV^−^ TSCC/BOTSCC (*p* = 0.0061). The frequency of mutated PIK3CA was similar to that in HPV^+^ TSCC/BOTSCC.

### Clinical response in relation to variant genes indicates a role of FGFR3

Survival analysis was performed for the 277 HPV^+^ TSCC/BOTSCC patients treated with curative intent for genes with mutations in > 6% of the cases i.e. TP53, PIK3CA, FGFR3, FBXW7 and of which three (TP53, FGFR3 and PIK3CA) are potentially targetable. A significant correlation to clinical response was found for variants in FGFR3, but for none of the other tested genes.

**Figure 2:**
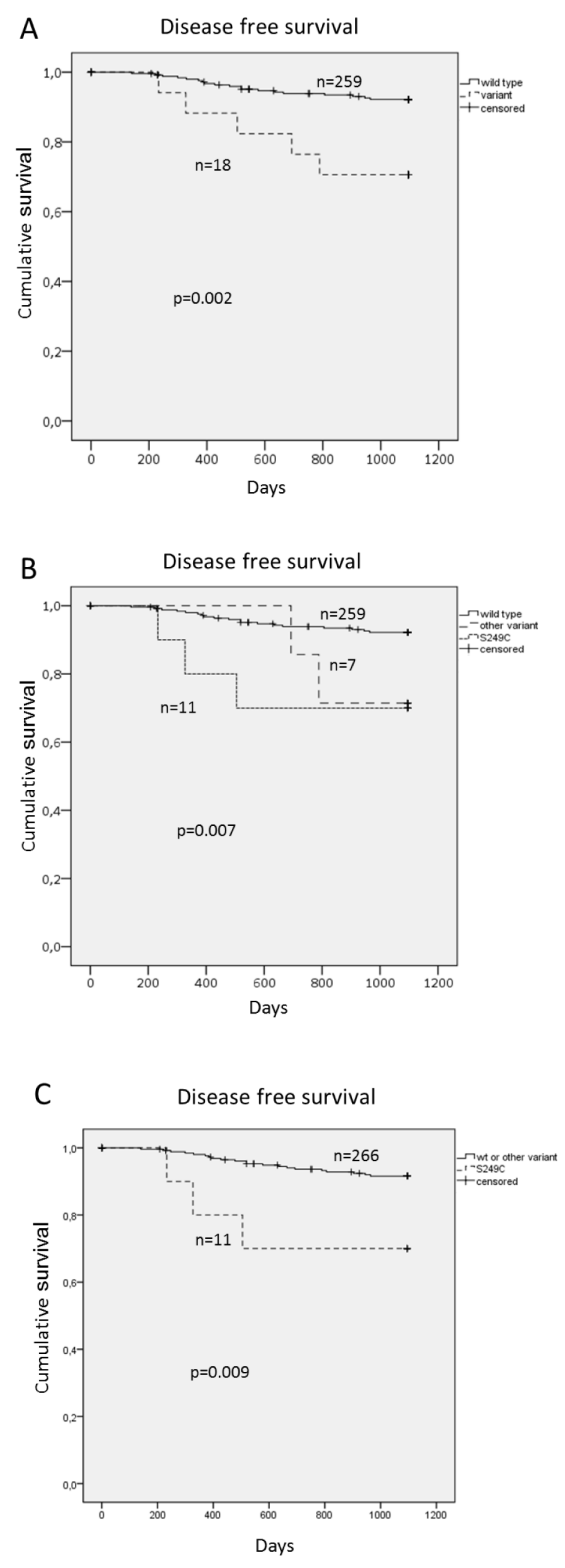
Disease free survival (DFS) for patients with HPV **^+^** TSCC/BOTSCC stratified for presence of FGFR3 variants. Cumulative DFS for HPV^+^ TSCC/BOTSCC: **A**. Stratified for any FGFR3 variant in relation to those with wild type FGFR3. **B**. Stratified in three categories, those with wild type FGFR3, those with the S249C variant and those with any other variant. **C**. Stratified between those with the S249C variant and those with wild type or other variants.

Of note, patients with HPV^+^ TSCC/BOTSCC with mutations in FGFR3 had a worse clinical outcome, depicted as 3-year disease free survival (DFS) (*p* = 0.002) (Figure 2A). Specific data on the FGFR3 mutations are presented in Supplementary Table S1. FGFR3 has previously been reported to be mutated in HPV^+^ HNSCC and the common specific variant of FGFR3, S249C, is a putative treatment target in urinary bladder cancer [31, 32]. This was also the most common FGFR3 variant in our study and was further investigated. Among the 19 HPV^+^ TSCC/BOTSCC patients treated with curative intent and with mutations in the FGFR3 gene, 11 carried the S249C variant. When HPV^+^ TSCC/BOTSCC cases were divided into those carrying the S249C variant, as compared to those with other FGFR3 variants and those with non-mutated FGFR3, patients with tumors carrying S249C had a significantly worse 3-year DFS (*p* = 0.009) (Figure 2B). When HPV^+^ TSCC/BOTSCC was stratified into three strata, those carrying the S249C variant, those with other FGFR3 variants and the rest of the cases, patients with tumors carrying S249C had a significantly shorter 3-year DFS (*p* = 0.007) (Figure 2C). Presence of FGFR3 mutations were also evaluated in relation to overall survival in HPV^+^TSCC/BOTSCC, but here statistical significance was not obtained (data not shown).

Survival analysis was also performed for the 43 HPV^−^ TSCC/OPSCC cases and for the 19 HPV^+^ HNCUP, where patients were treated with curative intent, for TP53, but not for any other mutations since the other cases were so few. No significant correlations were disclosed between TP53 and clinical outcome for either HPV^−^ TSCC/BOTSCC or HPV^+^ HNCUP.

## DISCUSSION

In this report, hotspot mutation regions of 50 cancer related genes were analyzed by NGS in 279 HPV^+^ and 46 HPV^−^ TSCC/BOTSCC and 19 HPV^+^ HNCUP. Commonly mutated variants were found in PIK3CA, TP53, FGFR3, FBXW7, PTEN and CDKN2A in HPV^+^ TSCC/BOTSCC, while in HPV^−^ TSCC/BOTSCC, mutations were most frequently found in TP53, PIK3CA and IDH2. HPV^+^ HNCUP showed mutations most often in TP53, PIK3CA and CDKN2A. The numbers of mutations per tumor were fewer in HPV^+^ TSCC/BOTSCC and HNCUP as compared to HPV^−^ tumors. PIK3CA was most frequently mutated in HPV^+^ TSCC/BOTSCC, while TP53 was the most commonly mutated gene in HPV^−^ TSCC/BOTSCC. The mutation pattern in HPV^+^ HNCUP, e.g. with regard to frequency of PIK3CA mutations, was similar to that of HPV^+^ TSCC/BOTSCC, although the frequency of TP53 mutations was a bit higher than that of HPV^+^ TSCC/BOTSCC. Of the variants tested, only FGFR3 variants correlated to clinical outcome in HPV^+^ TSCC/BOTSCC, specified as 3-year DFS, and especially those with the FGFR3 S249C variant had significantly worse prognosis.

The distribution of the various variants, and especially that PIK3CA mutations were more common in HPV^+^ TSCC/BOTSCC than in HPV^−^ TSCC/BOTSCC and vice versa for TP53 was in line with several earlier reports. [30, 31, 33]. However, in the present study the frequency of CDKN2A variants was clearly lower in HPV^−^ TSCC/BOTSCC as compared to some reports [30, 33–35]. Differences between studies might be due to dissimilarities in the targeted gene region, in the parameters for variant calling and filtering of data.

The finding that the total number of mutations in HPV^+^ TSCC/BOTSCC is lower than in HPV^−^ TSCC/BOTSCC is in concordance with e.g. Tinhofer et al., and could be due to the fact that high risk HPV provides active oncogenes E6 and E7 [30, 36]. Nevertheless, the total number of mutations found in HPV^+^ and HPV^−^ TSCC/BOTSCC in other regions needs to be analyzed further, since in this study targeted sequencing was performed using a pre-made cancer panel that covered certain areas of the selected genes. This can also explain some other differences between our study and other studies using larger panels and other experimental approaches, where they indicated larger numbers of mutations and fewer differences between HPV^+^ and HPV^−^ HNSCC [30, 31]. Still, when comparative genomic hybridization was performed, HPV^+^ TSCC and HNSCC respectively, were reported to have fewer gains and losses than HPV^−^ TSCC and HNSCC [37, 38].

To our knowledge, the mutational profile of HPV^+^ HNCUP has not been studied before and here we demonstrated that TP53, PIK3CA and CDKN2A were the most frequently altered genes in this subset of HNSCC. That HPV^+^ HNCUP had fewer mutations than HPV^−^ TSCC/BOTSCC, and a similar mutation frequency of PIK3CA to HPV+ TSCC/BOTSCC strengthens the hypothesis by others and us that HPV^+^ HNCUP originates from HPV^+^ OPSCC [4, 26–28]. Nonetheless, TP53 mutations in HPV^+^ HNCUP were more common than in HPV^+^ TSCC/BOTSCC, but less frequent than in HPV^−^ TSCC/BOTSCC. Thus, there were both similarities and differences between HPV^+^ HNCUP and HPV^+^ TSCC/BOTSCC. Whether, this is due to that HPV^+^ HNCUP should be regarded as HPV^+^ TSCC/BOTSCC metastasis, with additional genomic alterations, needs to be explored further. Notably, the HPV^+^ HNCUP group contained fewer never smokers than the HPV^+^ TSCC/BOTSCC group, which to some extent could explain the higher rate of TP53 mutations in the former. Attempts were made to find correlations between commonly occurring variants within each group and clinical outcome. For HPV^+^ TSCC/BOTSCC, mutations in PIK3CA and TP53 did not correlate to clinical outcome, while notably mutations in FGFR3 and especially the S249C variant correlated to worse prognosis. Variants of FGFR3, including S249C have been reported previously in HPV^+^ HNSCC, but have to our knowledge not previously been correlated to clinical outcome in HPV^+^ TSCC/BOTSCC [30, 31, 33, 35]. The reported frequency of FGFR3 variants in HPV^+^ HNSCC varies between 0 and 24% [30, 31, 33, 35]. Notably, in bladder cancer the frequency of FGFR3 overexpression is much higher than the frequency of FGFR3 mutations, indicating that in HPV^+^ HNSCC more tumors may be affected than can be found in a mutational analysis [39]. There are reports describing that it is possible to target FGFR3, and specifically the S249C variant, e.g. in bladder cancer cell lines, opening an option for more personalized treatment in the future [32, 40]. Furthermore, although PIK3C and TP53 variants could not be observed to relate to clinical outcome in HPV^+^ TSCC/BOTSCC, the possibility still remains that they too could be targeted in the future and present a possibility for more personalized treatment.

In this investigation, mutation in TP53 did not correlate to clinical outcome in the HPV^−^ cancer group, which differs from a previous report, where mutated TP53 conferred worse outcome in HPV^−^ HNSCC [30]. There is no evident explanation for this discrepancy however there are differences in the choice of sequencing methods and sequencing gene panels as well as number and type of patients included for the analysis [30]. Furthermore, in this study TP53 mutations were defined as of moderate and of high impact and correlated to 3-year DFS, while in the other report the distinction was related to degree of hotspot missense TP53 mutations and correlated to locoregional recurrence [30]. Other mutations in HPV^−^ TSCC/BOTSCC, such as PIK3CA, were not analyzed in relation to clinical outcome due to the limited numbers of cases making it a target for further analysis. However, no such correlation was previously identified despite the larger number of HPV^−^ HNSCC cases in the work of Tinhofer et al [30].

There are limitations in this study in that only hotspot mutations of 50 oncogenes and tumor suppressor genes were analyzed, and that our inquiry was retrospective. Furthermore, only 19 HPV^+^ HNCUP cases were included. Nevertheless, some interesting findings were still observed.

To conclude, when studying hotspot mutations in 50 oncogenes and tumor suppressor genes, the frequency of mutations per tumor were similar and fewer in HPV^+^ TSCC/BOTSCC and HPV^+^ HNCUP, compared to, and differing from HPV^−^ TSCC/BOTSCC. Furthermore, for the first time specific FGFR3 mutations (S249C) in HPV^+^ TSCC/BOTSCC were disclosed as a significant risk for worse clinical outcome, also opening up for novel therapeutic options for these patients.

## MATERIALS AND METHODS

### Patients and tumor characteristics

Patients diagnosed with TSCC (ICD-10 code C09.0-9) or BOTSCC (ICD-10 code C01.9) or HNCUP/secondary and unspecified malignant neoplasm of lymph nodes of head, face and neck (ICD-10 C77.0) between 2000-2011 at Karolinska University Hospital were included in the study. Having an HPV^+^ TSCC/BOTSCC in this study was defined as having an HPV DNA positive tumor combined with overexpressing p16^INK4A^ (p16), and if not p16 positive, as in one case, instead expressing HPV16 E7, while HPV^−^ TSCC/BOTSCC was defined as having no HPV DNA. TSCC/BOTSCC not fulfilling these criteria, i.e. with dubious HPV status, were excluded from the analysis. By this definition this investigation initially included FFPE biopsy material from 297 HPV^+^ TSCC/BOTSCC and 51 HPV^−^ TSCC/BOTSCC. Data on presence or absence of HPV DNA and p16 expression and HPV16 mRNA expression in the biopsies were derived from previous studies [16, 22, 23]. HPV DNA and p16 data on the 20 HPV^+^ HNCUP were obtained from a previous report where all cases but two overexpressed p16 [4]. The study was performed according to permission 2009/1278-31/4 from the Ethical Committee at Karolinska Institutet.

### DNA extraction and analysis of HPV DNA and p16 overexpression

DNA was extracted and HPV DNA status was assayed by a PCR-based bead based multiplex assay on a MagPix instrument (Luminex Inc.) as described previously [41]. p16 expression was examined using the monoclonal antibody (mAb) clone JC8 (Santa Cruz Biotech, Santa Cruz, CA, USA) [5, 22, 24].

### Library preparation

Hotspot regions in cancer related genes were analyzed by targeted amplification by PCR using the Ion AMpliSeq Cancer Hotspot Panel v2 (CHPv2 - Thermo Fisher Scientific), which covers 2800 hotspots in the following 50 genes; ABL1, AKT1, ALK, APC, ATM, BRAF, CDH1, CDKN2A, CSF1R, CTNNB1, EGFR, ERBB2, ERBB4, EZH2, FBXW7, FGFR1, FGFR2, FGFR3, FLT3, GNA11, GNAQ, GNAS, HNF1A, HRAS, IDH1, IDH2, JAK2, JAK3, KDR, KIT, KRAS, MET, MLH1, MPL, NOTCH1, NPM1, NRAS, PDGFRA, PIK3CA, PTEN, PTPN11, RB1, RET, SMAD4, SMARCB1, SMO, SRC, STK11, TP53 and VHL.

Amplicon libraries were prepared according to the manufacturer's protocol. In brief, 10 ng of genomic DNA from tumor samples was measured by Qubit 2.0 Fluorometer (Thermo Fisher Scientific) and amplified in a single multiplex PCR reaction obtaining 207 amplicons with sizes ranging from 49 to 140 bp. Next, amplicons were treated with FuPa Reagent (Thermo Fisher Scientific) to partially digest the primers and phosphorylate the amplicon ends, and the products were ligated to the sequencing adapters with 96 unique Ion Xpress Barcodes (Thermo Fisher Scientific) according to the manufacturer's instructions. After AMPure beads purification (Beckman Coulter), all barcoded libraries were quantified by the Agilent 2100 Bioanalyser and Agilent High Sensitivity DNA Kit (Agilent Technologies). The final library concentrations were standardized to 100 pM in low Tris-EDTA (TE) buffer.

Emulsion PCR and enrichment were performed on an Ion One Touch System by using the Ion PI Hi-Q OT2 200 Kit (Thermo Fisher Scientific) according to the manufacturer's instructions. Lastly, 96 pooled samples were loaded on a Ion P1 chip and sequenced on the Ion Proton benchtop sequencing platform using the Ion PI Hi-Q Sequencing 200 Kit (Thermo Fisher Scientific).

The Ion Torrent Variant Caller Plugin v5.0 was used to align reads to the reference genome hg19.

### Variant calling

The Ion Torrent platform-specific pipeline software Torrent Suite used data from the initial Ion Proton runs to generate sequence reads, trim adapter sequences and filter and remove poor signal profile reads. Then, Torrent Suite Software v5.0, with a plug-in “variant caller v5.0” program (TVC), was used to call variants from the initial sequencing data. In order to eliminate base calling errors, several filtering steps were employed to generate final variant calling. First, variants samples with poor DNA quality were excluded, defined as < 80% on-target reads and/or mean coverage < 400 reads. Additional filters for variant calling were fixed at an average total coverage depth > 100, single variant coverage > 20, a variant allele frequency (VAF) between 5% and 90%. Furthermore, low impact variants were not retained, as well as germline variants, defined as variants with VAF > 1% in any of the population genome databases; 1000 genomes [42] (All1000genomes and Eur1000genomes) and ExAC [43] databases. Also, two additional common SNP/MNPs, present in the NCBI dbSNP [44] database but not present in the COSMIC database [45], were filtered out. Lastly, dubious variants were visually inspected using Integrative Genomics Viewer (IGV) software.

### Statistical analysis

Clinical outcome was measured as 3-year disease-free survival (DFS) from the date of diagnosis. An event was defined as recurrence in the disease. Patients who were never tumor-free were counted as having relapsed on day 0, while patients who died without prior recurrence were censored at that time-point. Statistical calculations were performed using IBM SPSS Statistics software (Version 23.0; IBM Corp., Armonk, NY, USA). Survival curves and 3-year DFS were calculated using the Kaplan-Meier method and differences in survival were tested using the log-rank test. Frequencies of variants in the different genes in Table 3 were compared by the Chi-square or Fisher's exact test. Two-sided p-values were reported for all analyses and p-values below 0.05 were considered as significant. Correlations were calculated between all mutated genes and HPV status, using Spearman's test. Any mutation in a gene was coded as 1 and absence of any mutation as 0. Significance values of correlations were calculated with the asymptotic t-approximation, using the cor. test function in R.

## SUPPLEMENTARY MATERIALS FIGURE AND TABLE


