# Supplementary material for: Targeted sequencing of tonsillar and base of tongue cancer and human papillomavirus positive unknown primary of the head and neck reveals prognostic effects of mutated FGFR3
Source: Oncotarget. 2017 Feb 9;8(21):35339–50. doi: 10.18632/oncotarget.15240 (PMC5471059; doi:10.18632/oncotarget.15240)
Supplement: Supplementary file 1 [file oncotarget-08-35339-s001.pdf]

# Targeted sequencing of tonsillar and base of tongue cancer and human papillomavirus positive unknown primary of the head and neck reveals prognostic effects of mutated FGFR3

## Supplementary Material

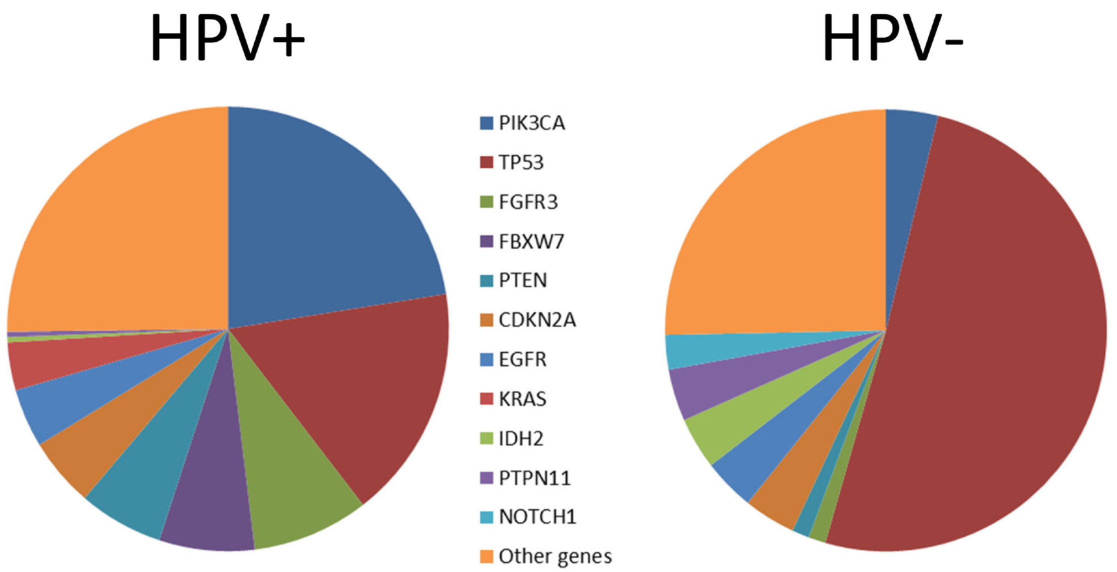


**Supplementary Figure S1: Pie charts illustrating the relative distribution of detected mutations among the 50 analyzed genes in $HPV^+$ and $HPV^-$ TSCC/BOTSCC respectively.**

**Supplementary Table S1: Complete list of FGFR3 variants in HPV$^{+}$ and HPV$^{-}$ TSCC/BOTSCC**

| Sample no | Diagnosis | HPV status | Position on chr# 4 | Reference base | Alternate base | Cosmic | dbSNP | Allele frequency |
|---|---|---|---|---|---|---|---|---|
| 2 | BOTSCC | positive | 1803564 | C | T | COSM714 | | 0.439 |
| 6 | BOTSCC | negative | 1803568 | C | G | COSM715 | | 0.321 |
| 10 | BOTSCC | positive | 1808979 | C | T | | | 0.293 |
| 10 | BOTSCC | positive | 1803568 | C | G | COSM715 | | 0.253 |
| 39 | BOTSCC | positive | 1803564 | C | T | COSM714 | | 0.493 |
| 42 | BOTSCC | positive | 1807889 | A | G | COSM726;COSM719 | | 0.333 |
| 49 | BOTSCC | positive | 1803568 | C | G | COSM715 | | 0.385 |
| 92 | TSCC | positive | 1803568 | C | G | COSM715 | | 0.153 |
| 108 | TSCC | positive | 1803568 | C | G | COSM715 | | 0.054 |
| 143 | TSCC | positive | 1803568 | C | G | COSM715 | | 0.163 |
| 152 | TSCC | positive | 1806131 | T | C | COSM724 | rs17881656 | 0.732 |
| 164 | TSCC | positive | 1803568 | C | G | COSM715 | | 0.230 |
| 170 | TSCC | positive | 1803568 | C | G | COSM715 | | 0.143 |
| 199 | TSCC | positive | 1808324 | G | A | | | 0.404 |
| 309 | TSCC | positive | 1806119 | G | A | COSM24842 | | 0.416 |
| 309 | TSCC | positive | 1803564 | C | T | COSM714 | | 0.059 |
| 314 | TSCC | positive | 1803568 | C | G | COSM715 | | 0.346 |
| 321 | TSCC | positive | 1803568 | C | G | COSM715 | | 0.464 |
| 363 | TSCC | positive | 1806131 | T | C | COSM724 | rs17881656 | 0.393 |
| 386 | BOTSCC | positive | 1808898 | C | T | | | 0.289 |
| 572 | TSCC | positive | 1803568 | C | G | COSM715 | | 0.173 |
| 594 | TSCC | positive | 1803568 | C | G | COSM715 | | 0.600 |
| 732 | BOTSCC | positive | 1803568 | C | G | COSM715 | | 0.661 |
